# Evaluation of the Keeogo exoskeleton for assisting ambulatory activities in people with multiple sclerosis: an open-label, randomized, cross-over trial

**DOI:** 10.1186/s12984-018-0468-6

**Published:** 2018-12-12

**Authors:** Chris A. McGibbon, Andrew Sexton, Arun Jayaraman, Susan Deems-Dluhy, Pearl Gryfe, Alison Novak, Tilak Dutta, Eric Fabara, Catherine Adans-Dester, Paolo Bonato

**Affiliations:** 1 Institute of Biomedical Engineering, University of New Brunswick, Fredericton, NB Canada; 2 Faculty of Kinesiology, University of New Brunswick, Fredericton, NB Canada; 3 Shirley Ryan AbilityLab / Rehabilitation Institute of Chicago, Chicago, IL USA; 4 Assistive Technology Clinic, Toronto, ON Canada; 5 Toronto Rehabilitation Institute, Toronto, ON Canada; 6 Institute of Biomaterials and Biomedical Engineering, University of Toronto, Toronto, ON Canada; 7 Department of Physical Medicine and Rehabilitation, Harvard Medical School, Spaulding Rehabilitation Hospital, Charlestown, MA USA

**Keywords:** Mobility, Powered orthosis, Neurological disorder, Ambulation aid, Home mobility aid

## Abstract

**Background:**

Although physical activity and exercise is known to benefit people with multiple sclerosis (MS), the ability of these individuals to participate in such interventions is difficult due to the mobility impairments caused by the disease. Keeogo is a lower-extremity powered exoskeleton that may be a potential solution for enabling people with MS to benefit from physical activity and exercise.

**Methods:**

An open-label, randomized, cross-over trial was used to examine the immediate performance effects when using the device, and the potential benefits of using the device in a home setting for 2 weeks. Clinical performance tests with and without the device included the 6 min walk test, timed up and go test and the 10-step stair test (up and down). An activity monitor was also used to measure physical activity at home, and a patient-reported questionnaire was used to determine the amount and extent of home use. Generalized linear models were used to test for trial effects, and correlation analysis used to examine relationships between trial effects and usage.

**Results:**

Twenty-nine patients with MS participated. All measures showed small decrements in performance while wearing the device compared to not wearing the device. However, significant improvements in unassisted (Rehab effect) performance were found after using the device at home for 2 weeks, compared to 2 weeks at home without the device, and participants improved their ability to use the device over the trial period (Training effect). Rehab and Training effects were related to the self-reported extent that participants used Keeogo at home.

**Conclusions:**

Keeogo appears to deliver an exercise-mediated benefit to individuals with MS that improved their unassisted gait endurance and stair climbing ability. Keeogo might be a useful tool for delivering physical activity interventions to individuals with mobility impairment due to MS.

**Trial registration:**

ClinicalTrials.gov: NCT02904382. Registered 19 September 2016 - Retrospectively registered.

## Background

Multiple sclerosis (MS) is a progressive neurological disorder that can profoundly impact mobility, independence and quality of life [1]. Muscle fatigue, pain and weakness [2] are all factors that contribute to patients’ limitations in ambulatory activity. Assistive devices such as canes, walkers and rollators can help with ambulatory activity [3, 4], but are only meant to assist and not to improve physical endurance of the user. Exercise interventions aimed at improving physical endurance in people with MS have been moderately successful in clinical trials [5–9]. However, delivery of such interventions in a clinical setting is expensive and resource intensive, and mobility difficulties play a significant role in why many patients with MS do not, or cannot, seek physical therapy services [10]. There is a need for innovative solutions for improving exercise capacity of individuals with MS in order to benefit from available interventions.

Keeogo (B-Temia Inc., Quebec City, Canada) is a lower-extremity powered exoskeleton intended to assist with ambulatory activities of individuals with MS and other neurological or musculoskeletal conditions that cause mobility impairment. The device could represent a possible solution to assisting individuals in the home and community, and for delivering physical activity-based exercise interventions to individuals who otherwise would have difficulty performing such activities.

Although other over-ground exoskeletons are commercially available, such as Indego, Ekso and ReWalk [11, 12], these are powered hip and knee devices meant to be used in a rehabilitation setting primarily and function to assist patients’ entire gait cycle during gait training. Recently (c.2014), they have moved into the personal mobility market for individuals with spinal cord injury for level ground walking only. In contrast, Keeogo does not initiate or terminate movement, rather it actively assists or resists during key phases of movement, such as stance and swing phase of gait or to assist when rising from a chair as well as functional activities such as stair climbing, jogging etc. Keeogo has motors only at the knees, and does not support or constrain the feet, pelvis or torso (users must be ambulatory). Although it is considerably lighter and slimmer than other commercial devices, people with MS are highly susceptible to fatigue [13] and are at increased risk of falling [14, 15]. Therefore it is critical to evaluate these technologies for their benefits and risks.

To evaluate if there are benefits of using Keeogo for the MS population, we designed and executed an open-label, randomized, cross-over trial to examine functional level outcomes when using Keeogo as an ambulatory assist device in the clinic, and in the home and community environment. The experimental design allowed us to analyze the immediate performance effects *while* using the device, and the acquired performance effects *from* using the device. Self-report disability instruments, usability analysis, and safety outcomes were also acquired. These latter outcomes are in preparation for publication.

The purpose of this paper was to evaluate the effects of the Keeogo exoskeleton on the physical performance of individuals with MS in a clinical setting, their physical activity levels and other potential benefits while using it in a home setting. Specifically, we sought answers to the following questions.

1. Do individuals with MS improve their physical performance while wearing Keeogo (Performance effect)?
2. Do individuals with MS improve their physical activity levels while using Keeogo at home for 2 weeks (Activity effect)?
3. Do individuals with MS improve their physical performance without Keeogo after using Keeogo at home for 2 week (Rehab effect)?
4. Do individuals with MS improve their physical performance with Keeogo after using Keeogo at home for 2 week (Training effect)?
5. Are these effects related to one another?

## Methods

### Study design

**Fig. 1:**
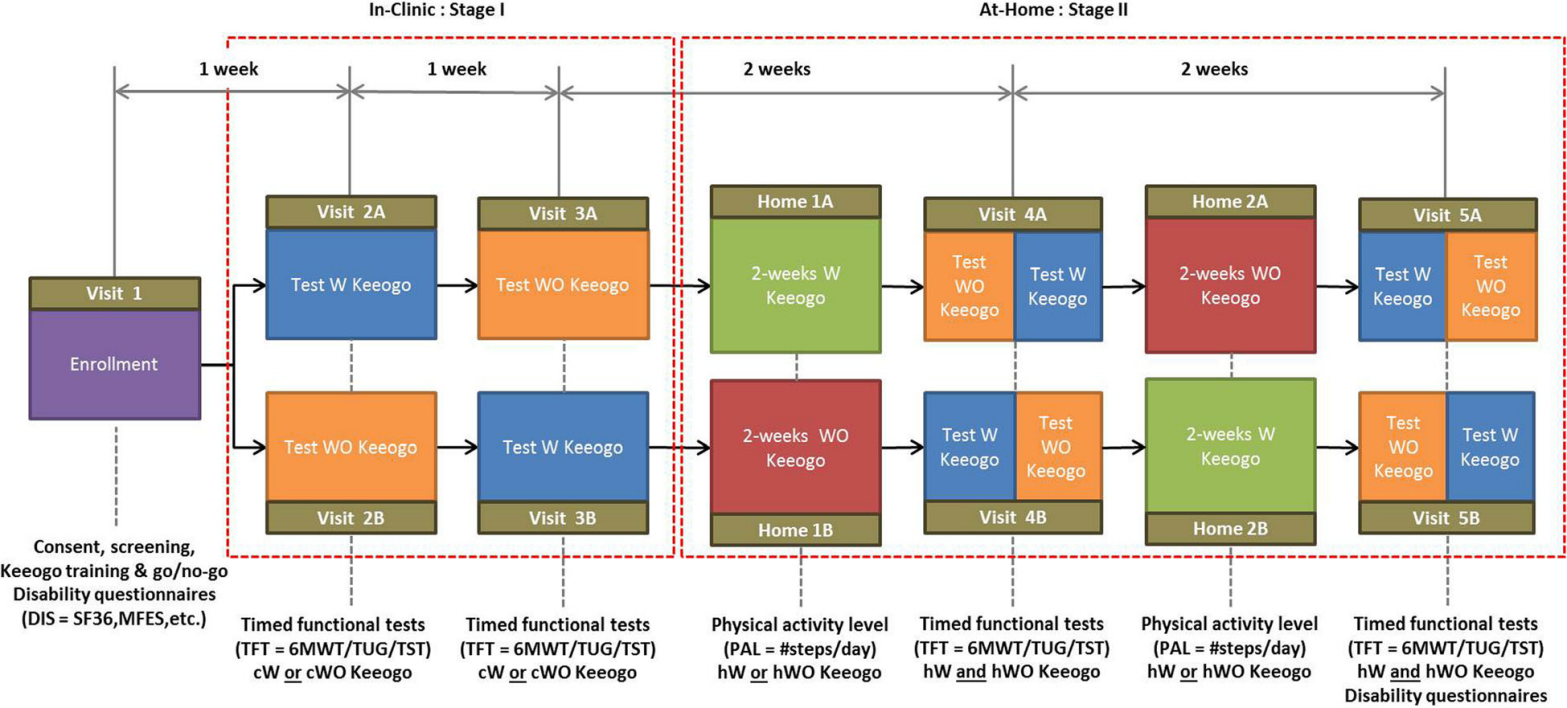
Trial schema. After enrollment (Visit 1) participants are allocated to test sequences A or B. Visits 2 and 3 form Stage I of the trial, which was to quantify the immediate and naïve effects of Keeogo™ on physical performance tests in a clinical setting by comparing test performance with (cW) and without (cWO) using Keeogo™. During Stage II of the trial, participants used Keeogo™ at home for 2 weeks (hW) and for 2-week without (hWO) Keeogo™. Participants were monitored all 4 weeks using an Actigraph activity monitor, in order to quantify the effects of Keeogo™ on physical activity levels. Clinical testing at mid-way (Visit 4) and post (Visit 5) was used to quantify any changes in participants physical performance with (cW) or without (cWO) Keeogo™ resulting from home usage

This was a multicentre, open-label, randomized, cross-over trial. The trial schema is illustrated in Fig. 1.

### Study sites

Four centres participated in patient recruitment and testing: Shirley Ryan AbilityLab/ Rehabilitation Institute of Chicago (Chicago, IL), Spaulding Rehabilitation Hospital (Charlestown, MA), Toronto Rehabilitation Institute (Toronto, ON) and the Assistive Technology Clinic (Toronto, ON). The study was led by the University of New Brunswick (Fredericton, NB). All sites received ethics approval from their respective IRB/REB.

### Participants

Participants with a diagnosis of multiple sclerosis (MS) were recruited between September 2015 and February 2017. Site staff (e.g. research coordinators) recruited participants through their local clinic(s) and partnering hospitals. All participants provided signed informed consent prior to enrollment.

Inclusion and exclusion criteria are shown in Table 1. Participants were included based on their clinical presentation rather than MS phenotype (remitting/relapsing, primary progressive, etc.). Clinical presentation was evaluated based on disease severity (≤6.5 on the Kurtzke Expanded Disability Severity Scale (EDSS) [16], physical function (ability to perform a 25 m walk test and 10 step stair test without other human assistance), cognitive function (score > 23 on the Mini-mental State Examination (MMSE) [17]), and spasticity (score < 3 for any leg joint on the Modified Ashworth Scale (MAS) [18]). Furthermore, participants had to successfully complete a Keeogo training session and competency test prior to being enrolled. All screening assessments were conducted by site staff.

**Table 1:** Inclusion and exclusion criteria

Inclusion Criteria	
Adult 21 or over diagnosed > 1 yr. ago with multiple sclerosis	
Able to read and understand informed consent form and study instructions	
Waist and leg circumference and lower extremity lengths appropriate for a comfortable and safe fit in the Keeogo device	
Able to walk 25 m without stopping, without human assistance, using assistive devices and ankle-foot-orthoses, as necessary	
Can complete a 10 step stair test	
Score > 23 on the Mini-Mental State Examination	
Modified Ashworth Score (MAS) < 3 for knee or hip, and < 3 for ankle if no AFO is used	
Recent (< 12mo) Kurtz Expanded Disability Status Score (EDSS) evaluation on record, with EDSS ≤6.5	
Exclusion Criteria	
Legally blind	
Pregnant or lactating	
Skin condition that contraindicates use of orthotics or support braces	
Recent (< 6 mo) lower-body hospitalizations or active treatments due a joint, muscle, bone, nerve or vascular injury or condition	
Scheduled for major surgery within next 4 months	
Lower-extremity amputation above or below the knee	
Have uncontrolled hypertension	
Recent (< 1 year) heart attack	
Have uncontrolled diabetes	
Diagnosed with other health condition(s) that affect mobility and balance, including chronic obstructive pulmonary disease; peripheral arterial disease; vestibular disorders; cerebellar disease; cerebral palsy; muscular dystrophy; spinal cord injury; stroke or other brain injury.	

Demographic data collected consisted of age, sex, ethnic background, and native language, as well as details of living environment (dwelling type, location, etc.)..

### Interventions and assessments

#### Randomization

Randomized ID code lists (<sequence group> < site # > <subject #>) were generated (by C.M.) using block randomization across test sites with equal numbers of A and B assignments. Randomization tables and seeds were generated using the online tool at “randomization.com”. Randomization ID charts were then provided to each site, where enrollment was carried out by site research staff. Participants at each site were assigned the first available ID code at screening. Blinding was not possible in this trial.

#### Experimental device

**Fig. 2:**
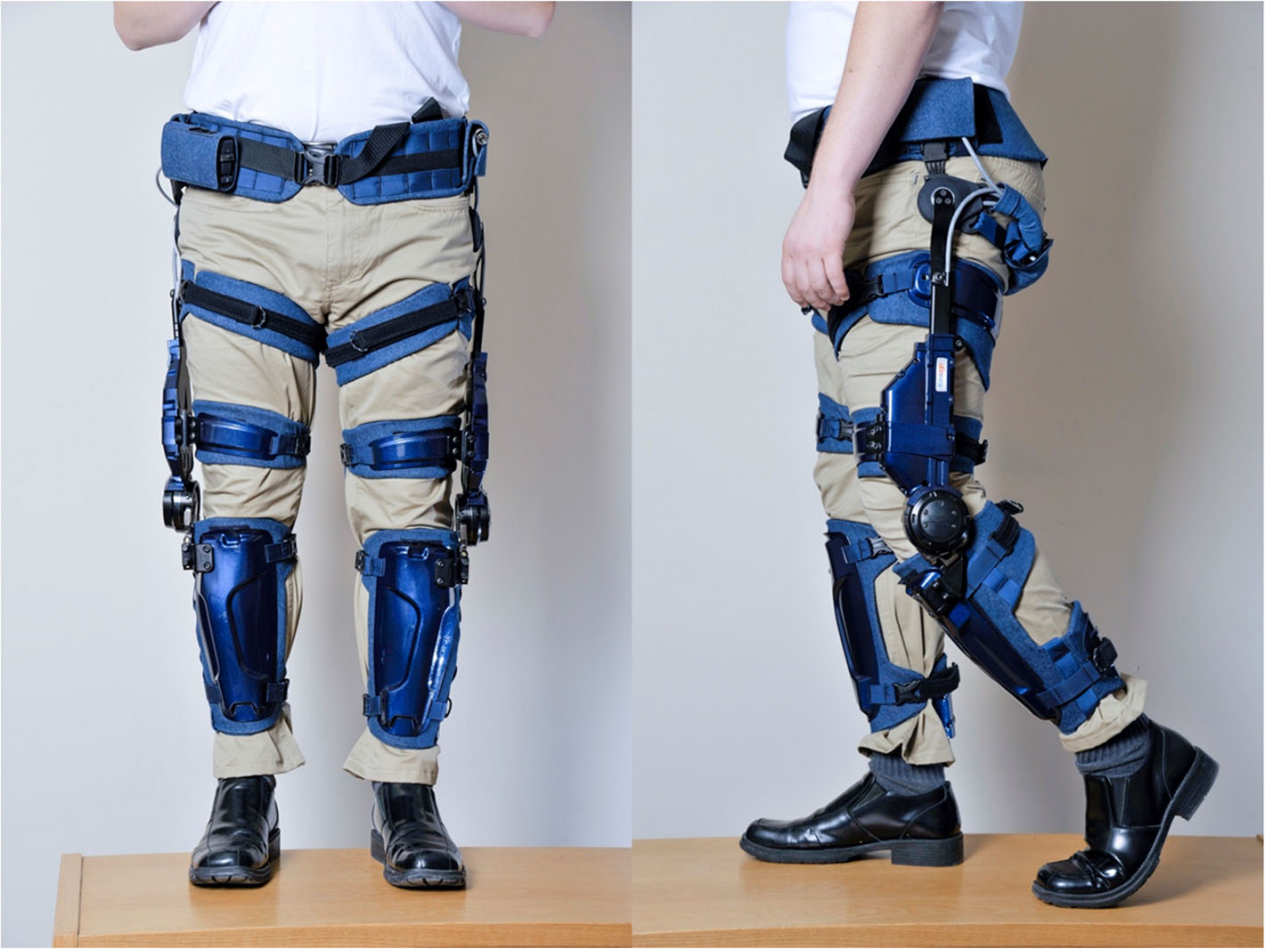
Keeogo™ Dermoskeleton by B-Temia Inc., Quebec, Canada. The device is worn over regular clothing. Motors are located at the knees. The device is secured to the limbs with shank and thigh cuffs, and also suspended from the waist belt and chariot system that allows the hip to rotate freely

The Keeogo exoskeleton orthotically fits to the user’s limbs and is intended to provide ambulatory assistance to individuals with gait impairment. It consists of bilateral motors for assisting left and right knees, a pelvis belt and chariot system for suspending the device, and thigh and shank cuffs for attaching the exoskeleton “links” to the user (Fig. 2). The controller recognizes standing, gait (walking, jogging and running full speed), stair ascent and descent, chair rising and sitting, squatting, lunging, and other locomotor tasks. The knee motor provides assistance to the user during specific phases of the task but does not initiate or terminate movements, while the hip motion of the device is passive. The shin cuffs can accommodate most ankle-foot orthoses, and the device can be used in conjunction with other assistive aids (cane, crutches, walkers, rollators, wheel chairs, etc.). With the battery pack (worn on the hip), Keeogo has a total mass of 5.4 kg.

Site therapists fitted and trained all participants. This session consisted of first fitting the Keeogo device for the participant, by adjusting the thigh length, waist belt and leg cuffs, to achieve a good suspension and alignment. The device was left in “passive” mode, while the participant moved about, and the fitting was adjusted if required. Participants with ankle-foot orthoses (AFO) were fit while wearing it. The device was then “tuned” for the participant by adjusting various assist parameters, via a tablet connected to Keeogo. The device was then put into “active” mode and the participant practiced walking and standing from a crouch, and adjustments were made if required. The fitting and training session lasted approximately 1.5–2 h for all participants.

The participant then completed the Keeogo competency test which tested: 1) walking, 2) turning, 3) get in and out of chair, 4) crouching and rising, and 5) going up and down stairs. The goal was to enroll only those who could, upon first use, use the technology in a demonstrably safe manner for activities they could be performing in the home and community. An inability to complete any one of the above activities after a reasonable number of attempts was deemed ineligible to participate in the trial. If successful, the participant was enrolled and all fitting and tuning parameters were documented. Site therapists maintained tuning parameters on the tablet so they could be adjusted in the future if required.

#### Experimental design

The trial was executed in two sequential stages (I and II). The design used was a 2 × 2 cross-over approach where subjects act as their own controls. Each trial arm was composed of a series of clinical assessments (“in-clinic” tests) at four time points over a 6 week period to measure patient’s physical performance (via a battery of timed functional tests, *TFT*s) in a clinical setting while wearing (cW) Keeogo or without wearing (cWO) Keeogo, where the order of cW and cWO conditions at each assessment was predetermined by group allocation (A or B). For each repetition of the TFTs, the participants rested for a minimum of 2 min and a maximum of 8 min between tests; between sets of tests, participants rested for a minimum of 10 min.

Interleaved within the clinical assessment schema was the “at-home” study, where participants’ physical activity levels (*PAL* = normalized step/day counts) were monitored at home for two sequential 2-week periods: at home with (hW) Keeogo and at home without (hWO) Keeogo, also ordered according to group allocation (ie. as a 2 × 2 cross-over). Clinic assessments (cW,cWO) were scheduled before, in between, and after, the two at-home sessions, as shown in Fig. 1, again using a testing sequence that counter balanced the order of W and WO conditions for both the clinic and home factors.

#### Outcomes measures

Efficacy was assessed with primary outcomes to measure performance change on timed functional tests (*TFT*) with and without wearing Keeogo, and physical activity levels (*PAL*) with and without using Keeogo at home.

##### Timed functional tests

*6 Minute Walk Test*: The 6MWT (distance in meters) is used to evaluate gait function and endurance [19], and has been reported as reliable and/or valid in many patient populations including those with MS [20]. Participants completed the test along a 25 m walkway. A stop watch was used to start and end the test. The observer counted the number of 25 m lengths achieved by the participant and then measured the distance of the last length to arrive at a total distance traversed during the 6 min walk. The test was repeated ν times (where ν is described below). For this test, higher scores (distance walked) means better gait endurance.

*Timed Up-and-Go:* The TUG test (time in seconds) is a ubiquitous clinical test of generalized mobility function [21] with well-known psychometric properties in numerous patient populations including patients with MS [22, 23]. A 3 m TUG test was used and followed the standard protocol outlined by Podsiadlo and Richardson [21]. Total time was recorded with a stop watch. The test was repeated ν times. For this test, lower scores (time to complete) means better generalized mobility.

*Timed Stair Test*: The TST (time in seconds) is a lesser used test of physical function in the clinic [24] but was selected for this study to evaluate how well participants could negotiate stairs when using the Keeogo. Participants first ascended, then descended, a 10-step stair case (e.g. from landing to landing in the stair well of the building). Time to ascend (TSTup) and descend (TSTdn) was measured separately with a stop watch. The test was repeated ν times. For this test, lower scores means better stair climbing performance.

Number of repetitions within each TFT test was set at ν = 5 for the first two visits to the clinic, where TFT testing was done with one of the two device conditions (cW,cWO), and ν = 3 for the last two visits where TFT testing was done for both device conditions. The different number of repetitions for the first two and last two visits was done to maintain similar length testing sessions. Repeated measures were used to get within-subjects variability for MDC calculations.

Order of testing within a session was TST, 6 MW and then TUG. This order was selected as it was decided in the design phase that should testing be too physically demanding for some participants, the TUG would be preferable to lose. The 6 MW test was expected to be the most fatiguing and therefore was placed after the TST. It was decided a fixed order was safer and more manageable for the clinical teams collecting data.

##### Physical activity level

Participants wore the Actigraph GT3X (ActigraphCorp, Pensacola, FL) activity monitor for two sequential 2-week periods (4 weeks in total) during the day, removing it only when sleeping or bathing. The device was set-up by study staff prior to participants taking it home, and data was downloaded after each 2-week period. Participants were instructed to wear the device on their hip. Two files were extracted for each at-home period: the 60 s epoch files containing step counts/min and the wear time file containing the number of minutes the device was worn each 24 h period. From these files the following metrics were calculated:

*Raw and adjusted mean steps per day:* SPDraw was found by summing counts in epochs of 60 s to get a total step count per 24 h period and averaging the daily totals over the number of days worn. However, wearing the monitor more will naturally capture more steps, and patients may vary in how much they actually wear the monitor (e.g. forgetting to don, variability in sleeping and bathing habits, etc.). Therefore, in order to control for amount of monitor wear, daily SPDraw was divided by wear time T (total time in minutes) to get average step/min, and multiplied by 16 h*60 min/hour (960 min) to represent a 16 h-wear equivalent, or SPDadj = 960*SPDraw/T. For this metric, higher mean SPD counts means higher levels of physical activity was performed during the monitoring period.

##### Amount and extent of use

*Keeogo Usability Survey:* As an exploratory tool, the KUS is a 74-item survey designed to measure amount of use, ease of use, comfort, satisfaction and safety. For the present analysis we used data from two sections of the KUS to quantify amount and extent of use of Keeogo in the home setting.

*Amount of use* (AMT) of Keeogo during the 2-week period at home was found from combining three items each on a 5-point scale: “Overall, how often did you use Keeogo?”, “On average, how would you describe your daily use of Keeogo?” and “On a day you used Keeogo the most, how long did you use it?” Responses to these items were summed and converted to a 0–100 scale.

*Extent of use* (EXT) measured whether more or less than usual activity was performed when using Keeogo, and was found by combining nine items that asked “Did you do more or less activity than you usually do?” with respect to “Getting around inside home”, “Standing activities”, “Bending/crouching activities”, “Going up and down stairs”, “Getting in and out of chair”, “Getting around outside home”, “Walking activities”, “Social activities” and “Leisure activities”. Responses to these items were summed and converted to a 0–100 scale.

Finally, Daily Activity Logs were kept by participants that allowed us to estimate the amount of time (in hours) during the day each participant used Keeogo during the 14 day period with the device.

### Data analysis

#### Statistical power

This analysis used the 6MWT as the primary powering variable, although TUG, TST and PAL measures were also considered in the analysis. Effect size and minimal detectable change (MDC) values were estimated by examining data from the literature for the TFT [20, 22, 23, 25–30] and PAL [31–33] measures. The resulting parameters used to power the study are summarized in Table 2. For a 2 × 2 cross-over design, with one-tailed type-I error rate α = .05 and type-II error rate β = .20 (power = .80) a total of 25 participants would be required. Assuming a drop-out rate of 20% (5 subjects) a total of sample of 30 was planned. Power analysis was conducted using a publically available cross-over sample size calculator (http://hedwig.mgh.harvard.edu/sample_size/js/js_crossover_quant.html).

**Table 2:** Parameters used to power the study

Measurement		TFT			PAL
Parameters		6 MWT (m)	TST (s)	TUG (s)	Step/day
Within-subject SD, σ		30.1	1.62	1.59	698
Superiority margin, MDC		65.4	3.47	3.50	1582
Hypothesize change, Δ		87.4	5.47	4.80	2082
Effect, δ = Δ-MDC		22.0	2.00	1.30	500
Required sample	N	25	10	26	26

*MDC* Minimal detectable change, *TFT* Timed functional test

*TUG* Timed up and go test, *6 MWT* 6 min walk test, *TST* timed stair test

*PAL* Physical activity level measured with Actigraph monitor

#### Statistical analysis

##### Primary outcomes

*Performance Effect:* The purpose of this outcome was to quantify the performance change while wearing Keeogo during the *TFT*s, compared to not wearing Keeogo during the *TFT*s. First, *TFT* results were averaged across the ν repeated tests within a session. A 2 × 2 ANOVA with one repeating factor (device used in clinic/not used in clinic) and one independent factor (sequence group A/B) was used to compare test means, controlling for order effects, between cW and cWO conditions using data from the first two visits to the clinic.

*Activity Effect:* The purpose of this outcome was to quantify if daily stepping activity changed while using Keeogo at home compared to daily stepping activity while at home without Keeogo. *PAL* data were quantified from normalized step/day counts. A 2 × 2 ANOVA with one repeating factor (device used at home/not used at home) and one independent factor (sequence group A/B) was used to compare *PAL* outcomes, controlling for order effect, between hW and hWO conditions. Note that at the start of the at-home periods, both sequence groups will have received the same amount of exposure to Keeogo, and their exposure during clinical assessments mid-way through the home period was also balanced, as shown in Fig. 1.

Multiplicity was addressed by applying a Bonferonni correction to the significance level for main effects, adjusted for the four outcome measures (3 TFT measures and 1 PAL measure). Therefore the criteria for significance was α = .013.

##### Secondary outcomes

*Rehab and Training Effects:* The purpose of this outcome was to quantify if participants’ timed functional tests improved *from* using Keeogo at home for a two-week period (“rehab effect”), and to quantify if participant’s performance *when* using Keeogo improved after using it at home for 2 weeks (“training effect”). For the rehab effect, a 3 × 2 ANOVA was used to compare the *TFT* change scores for the cWO condition, before and after the 2 week period hW, to change scores for the 2 week hWO period. Similarly for the training effect, a 3 × 2 ANOVA was used to compare *TFT* change scores for the cW condition before and after hW and hWO .

Multiplicity was addressed by applying a Bonferonni correction to the significance level for main effects, adjusted for the three outcome measures (3 TFT measures). Therefore the criteria for significance for these secondary tests was α = .017.

##### Exploratory outcomes

Relationships between the above outcomes measures were studied with correlation analysis to determine if the various effects measured were related or independent. Relationships were also explored between the primary and secondary effects and usability metrics (AMT, EXT). These exploratory tests were conducted at α = .05.

## Results

**Fig. 3:**
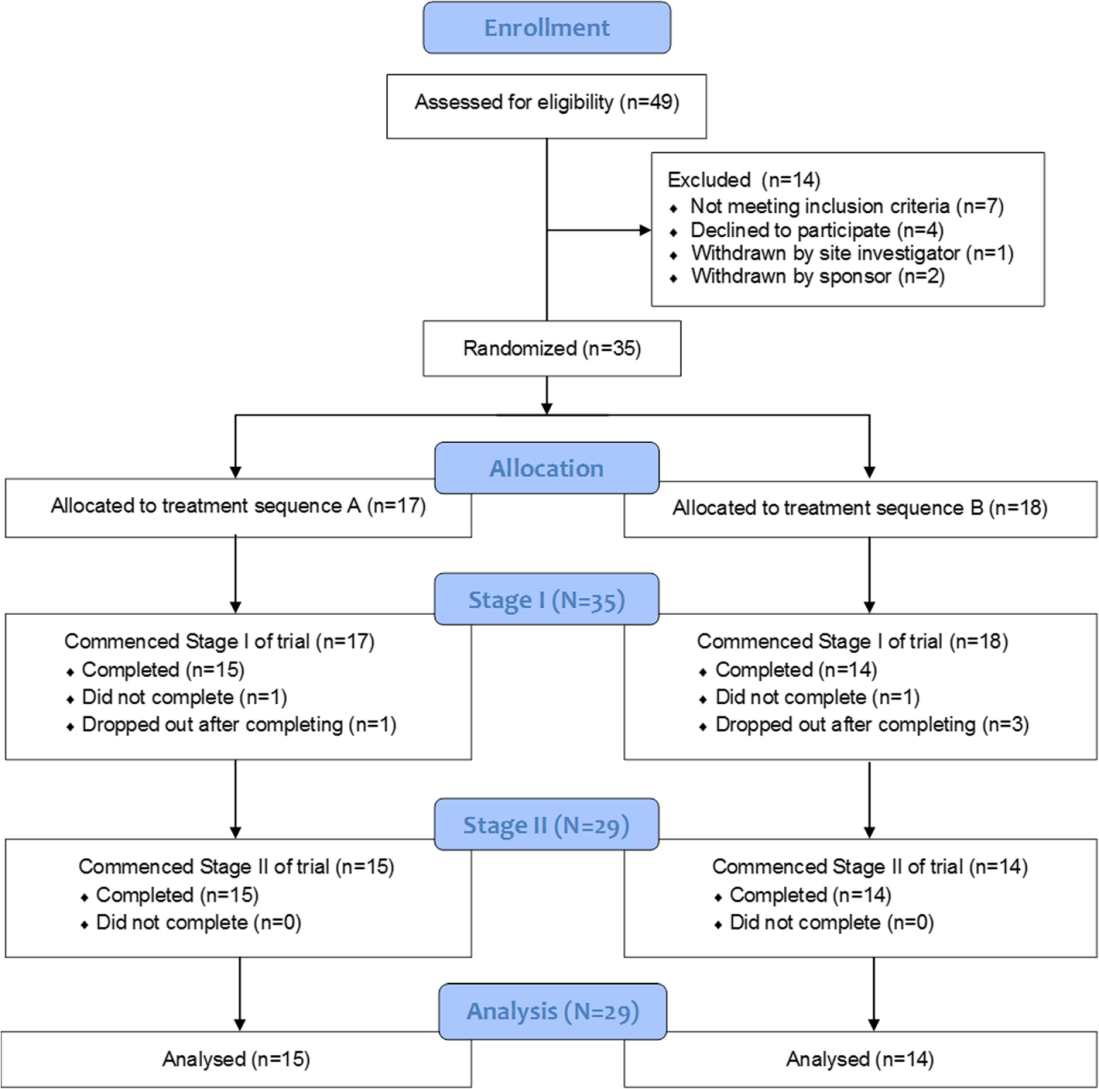
CONSORT flow diagram of trial recruitment

A CONSORT flow diagram is shown in Fig. 3. A total of 49 volunteers were screened for eligibility to participate in the study. Seven volunteers failed screening: five were not able to demonstrate the full set of core competencies required for using Keeogo, one did not pass the Mini Mental State Examination, and one did not meet fitting requirements. One volunteer that met the screening requirement was withdrawn prior to starting due to the site investigator’s medical opinion that participating would not be appropriate due to their frail state. Four volunteers that met the screening criteria declined to continue participation after consenting and were not tested. Two participants were enrolled but not able to be completed in time for this analysis, therefore withdrawn from the study by the Sponsor. Therefore, a total of 35 MS volunteers participated in the study.

Six of these 35 participants did not complete the study: Two dropped out during Stage I and were lost to follow-up (completed Visit 2 but not Visit 3) and four declined to continue participating after completing Stage I of the trial. Reasons consisted of being unable to comply with the time commitments of staying in the trial, worsening MS condition, or other unrelated injuries. There were no drop-outs during Stage II of the study. A total of 29 MS participants completed the study, with *n* = 15 allocated to sequence A and *n* = 14 allocated to sequence B. The average length of time in the trial was 43 ± 4 days. There were no differences between A and B groups in the duration of their trial.

Demographic and health status data are shown in Table 3. The sample was 49.2 ± 10.6 years old and 17 of the 29 participants were female. Body mass index (BMI) was low-normal. Mean EDSS score was 5.3 ± 1.3 and ranged between 4.0 and 6.5 (median score = 5). Average MAS score of most affected joint was .9 ± .8 (median score = 1), indicating spasticity levels were relatively low for this sample.

**Table 3:** Demographics and medical status, and comparison of these characteristics between cross-over groups, for completed Participants (*N* = 29)

		Total (N = 29)		A (N = 15)		B (N = 14)		A vs B stats^a^	
		Mean	SD	Mean	SD	Mean	SD	*t*	*p*
Age (years)		49.2	10.6	48.4	11.4	50.1	10.0	−.419	.679
Height (cm)		169.9	9.6	170.5	11.8	169.2	6.8	.347	.731
Weight (Kg)		72.9	17.5	74.1	16.6	71.6	19.0	.373	.712
BMI (Kg/m^2^)		25.2	5.4	25.5	5.1	24.9	6.0	.276	.784
EDSS (0–10)		5.3	1.3	5.5	1.1	5.0	1.5	1.01	.320
MAS (0–4)		0.9	0.8	0.9	0.8	0.9	0.8	−0.09	.926
MMSE (0–30)		29.1	1.2	28.7	1.4	29.6	0.6	−2.06	.049
		Count	%	Count	%	Count	%	χ^2^	*p*
Sex	Male	12	41.4	7	46.7	5	35.7		
	Female	17	58.6	8	53.3	9	64.3	.358	.550
Ethnicity	Not Hispanic	26	89.7	13	86.7	13	92.9		
	Hispanic	3	10.3	2	13.3	1	7.1	–	–
Race	White	21	72.4	9	60.0	12	85.7		
	Black	5	17.2	3	20.0	2	14.3		
	Asian	1	3.5	1	6.7	0	0.0		
	Other	2	6.9	2	13.3	0	0.0	–	–
Language	English	28	96.6	14	93.3	14	100.0		
	Other	1	3.4	1	6.7	0	0.0	–	–

^a^ Between A and B groups: Student’s *t*-test for scale variables and chi-square (χ^2^) test for Sex (insufficient cell size to test other categorical variables); degree of freedom (*df = 27*); significance, *p* with alpha level .05

*BMI* Body mass index, *EDSS* Expanded Disease Severity Scale, *MAS* Modified Ashworth Scale, *MMSE* Mini-mental State Examination

There were no statistically significant differences (*p* > .05) in sex, age, height, weight, BMI, EDSS score, and MAS score (of most involved joint) between participants randomized to A and B groups. Race, ethnicity and language could not be statistically compared due to too few cases in non-White/non-English categories. There was a difference in MMSE score between participants randomized to A and B groups (*p* = .049), but the difference was small and not likely important as both groups were in the high-normal range for the MMSE [17]. MMSE scores was 29.1 ± 1.2 (median score = 29).

### Primary outcomes

#### Performance effects

Data from participants’ first clinical performance testing with and without Keeogo was used to determine their ability to use the technology as a mobility assist device, and to quantify their baseline performance measures prior to the home evaluation. Means and standard deviations of TFT performance measures are summarized in Table 4.

**Table 4:** Performance measures for MS patients, allocated to sequences A and B, during various timed functional tests (*TFT*) in the clinic with and without wearing Keeogo™. These data reflect the naïve effects of using Keeogo™ during participants first set of performance tests (Visit 2 and 3)

*TFT* scores (Visit 2 and 3)	Total		A (N = 15)		B (N = 14)	
	Mean	SD	Mean	SD	Mean	SD
*6 MWT distance (m)*						
cWO	259.5	102.7	252.0	106.0	267.6	102.2
cW	236.8	100.6	224.7	96.1	249.8	107.2
δ	−22.7	32.1	−27.2	40.5	−17.8	19.8
*TST up time (s)*						
cWO	12.7	5.9	14.0	6.4	11.4	5.1
cW	17.6	8.8	19.3	7.6	15.7	9.8
δ	4.8	5.7	5.3	5.5	4.3	6.0
*TST down time (s)*						
cWO	13.1	7.0	14.2	7.2	11.9	6.8
cW	15.7	7.7	17.3	6.9	14.0	8.3
δ	2.6	4.2	3.1	3.9	2.1	4.6
*TUG (s)*						
cWO	16.2	5.8	17.6	6.7	14.8	4.4
cW	20.5	7.5	22.1	7.5	18.8	7.3
δ	4.3	4.3	4.6	4.7	4.0	4.1

*TFT* Timed functional test, *TUG* Timed up and go test, *6 MWT* 6 min walk test,

*TST* Timed stair test, *SD* Standard deviation

*cW* Clinical TFT with Keeogo, *cWO* Clinical TFT without Keeogo, *δ* cW-cWO

##### 6 minute walk test

For 6MWT there was a significant effect for device (*p* = .001), non-significant group effect (*p* = .581) and non-significant interaction effect (group×device: *p* = .424). 6MWTscores with Keeogo were lower than 6MWT scores without Keeogo.

##### Timed stair test

For the TST, stair up time (TST-up) showed a significant effect for device (*p* < .001), non-significant group effect (*p* = .219) and non-significant interaction effect (group×device: *p* = .647). Stair down results were similar, with TST-dn showing a significant effect for device (*p* = .002) and non-significant group effect (*p* = .281) and non-significant interaction effect (group×device: *p* = .505). TST scores with Keeogo were longer than TST scores without Keeogo.

##### Timed up and go test

For the TUG test, there was a significant effect for device (*p* < .001), non-significant group effect (*p* = .177) and non-significant interaction effect (group×device: *p* = .746). TUG scores with Keeogo were significantly longer than TUG scores without Keeogo.

#### Activity effects

Activity monitor data from participants’ at-home periods with and without Keeogo was used to determine if using the technology as a mobility assist device in the home would result in more physical activity, as quantified by step/day counts. These results are summarized in Table 5. Actigraph records were lost for one of the two home sessions for 5 participants due to technical failure or improper initiation of the device. Data in Table 5 show results for intention to treat (ITT) analysis and per-protocol (PP) analysis.

**Table 5:** Step counts for at home monitoring periods of 14+/−3 days W/O Keeogo and W Keeogo, randomized in sequence by group A (W-W/O) and B (W/O-W). Results for ITT analysis (N = 29) and PP analysis (*N* = 24)

Steps/day at home (ITT)	Total		A		B	
	Mean	SD	Mean	SD	Mean	SD
hWO	4425.1	2897.0	4523.9	3854.6	4326.3	1597.5
hW	4693.5	2996.0	4599.2	3811.0	4787.9	2024.9
δ	268.4	739.4	75.2	404.3	461.6	944.3
Steps/day at home (PP)	Mean	SD	Mean	SD	Mean	SD
hWO	4636.9	3096.4	4984.7	4220.5	4318.0	1658.3
hW	4963.6	3184.2	5080.4	4156.7	4856.5	2131.7
δ	326.8	806.9	95.8	458.6	538.5	1004.4

*ITT* Intention to treat analysis, *PP* Per-protocol analysis

*hW* Home PAL with Keeogo, *hWO* Home PAL without Keeogo, *δ* hW-hWO

##### Daily step count

The average daily step count for the sample was 4425 steps/day. Statistical testing (ITT) showed a non-significant interaction effect (home*group: *p* = .046) and non-significant main effects (home: *p* = .137; group: *p* = .786).

Both groups A and B increased their total daily step count when using Keeogo in the home compared to the period when not using Keeogo at home, but it failed to reach statistical significance (*p* = .046). The change was small with the overall effect being an average increase of 270 step/day, and much less than the calculated MDC of 1582 steps/day.

#### Rehab effects

**Fig. 4:**
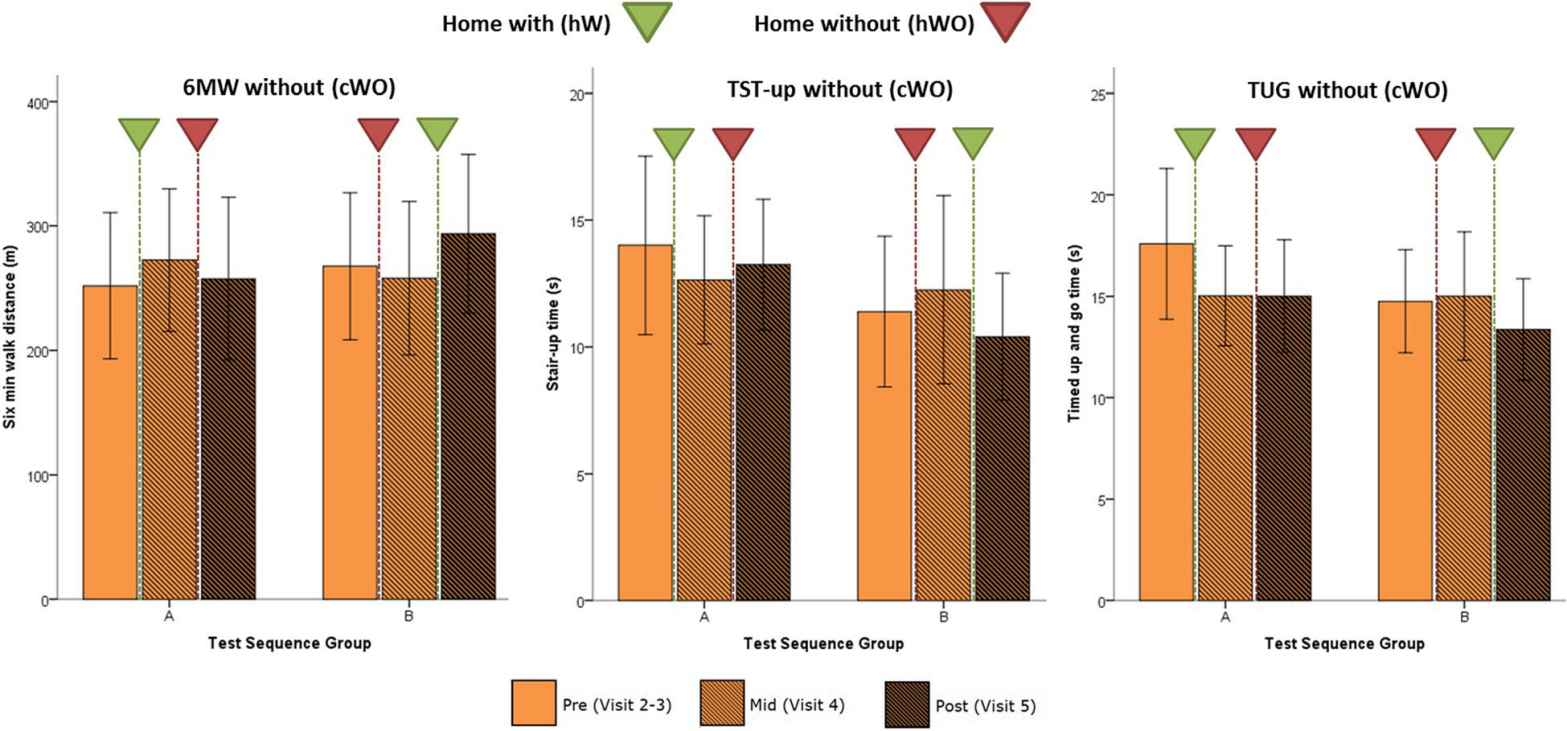
Rehab effect. TFT effects pre (light), mid-way (hatched), and post (dark) home trial, for 6 min walk (6 MWT) test, timed stair test (TST-up) and timed up and go test (TUG). Test results are without using Keeogo™ (cWO). Home trial interventions are shown by the inverted triangle, where the green triangle represents 2-weeks home with Keeogo (hW), and the red triangle represents 2-week home without Keeogo (hWO). Error bars represent 95% confidence intervals. Results are shown for test sequence groups A and B (note the sequence of hW and hWO differed for A and B). In all cases the largest improvements were seen after 2-weeks hW

Using Keeogo at home might impart functional benefits to the user that persist when not using the technology. These “plastic” effects are akin to a rehabilitation effect. TFT data without Keeogo (cWO) from participants’ visits before, mid-way, and following the two 2-week monitoring periods (hW,hWO) were used to quantify the rehab effect. These results are shown in Fig. 4. TFT scores for pre, mid and post-trial are shown for the cWO testing condition, with inverted triangles showing the hW (green) and hWO (red) period between each measurement session.

##### 6 minute walk test

A mixed ANOVA was conducted for 6MWT scores between visits (visit: pre, mid and post) and sequence group (group: A and B). The test yielded a significant visit effect (*p* = .006), non-significant group effect (*p* = .764) and a significant visit * group interaction (*p* < .001), indicating that test means were different across visits, but depended on sequence group (ie. active intervention). One-way ANOVA was then conducted within sequence groups. For group A, 6 MW test change scores for hW were greater than change scores for hWO, but failed to reach significance (*p* = .028). For group B, 6MWT change scores for hW were significantly greater (*p* = .004) than change scores for hWO.

These results show that the 2-week at-home period with Keeogo resulted in a statistically significant improvement in patients’ unassisted 6MWT distance, especially for group B. The overall effect was + 27.9 m which was significantly different from zero (95% CI, 15.1,40.6) but fell somewhat short of the MDC value for 6MWT (+ 65.4 m) in Table 2.

##### Timed stair test

A mixed ANOVA was conducted for TST up and down time scores between visits (visit: pre, mid and post) and sequence group (group: A and B). For stair up time, the test yielded a non-significant visit effect (*p* = .233), non-significant group effect (*p* = .283) and a non-significant visit * group interaction (*p* = .024). There were similarly no significant effects for stair down time. One-way ANOVA was then conducted within sequence groups for TST-up. For group A, TST-up change scores for hW were better than TST-up change scores for hWO, but the difference was not significant (*p* = .293), due to the high variability of the measures during participants first visit. For group B, TST-up change scores for hW were significantly better than change scores for hWO (*p* = .005).

These results show that the 2-week at-home period with Keeogo resulted in a statistically significant improvement in unassisted stair climbing performance, likewise especially for group B. The overall improvement in TST was − 3.06 s, which although statistically different from zero (95% CI: -5.60,-0.51), was short of the calculated MDC of − 3.47 s.

##### Timed up and go test

No statistically significant effects were found for visit * group interaction (*p* = .059) and therefore no post-hoc tests were conducted, however there was a significant effect of visit (*p* = .004).

These results suggest that the 3 m TUG performance improvements occurred more as a result of participating in the trial, and not directly related to home use. Nevertheless, the overall effect for the TUG was an improvement of − 2.12 s, which was significantly different from zero (95% CI: -3.65,-0.59) but less than the calculated MDC (− 3.50s).

#### Training effect

**Fig. 5:**
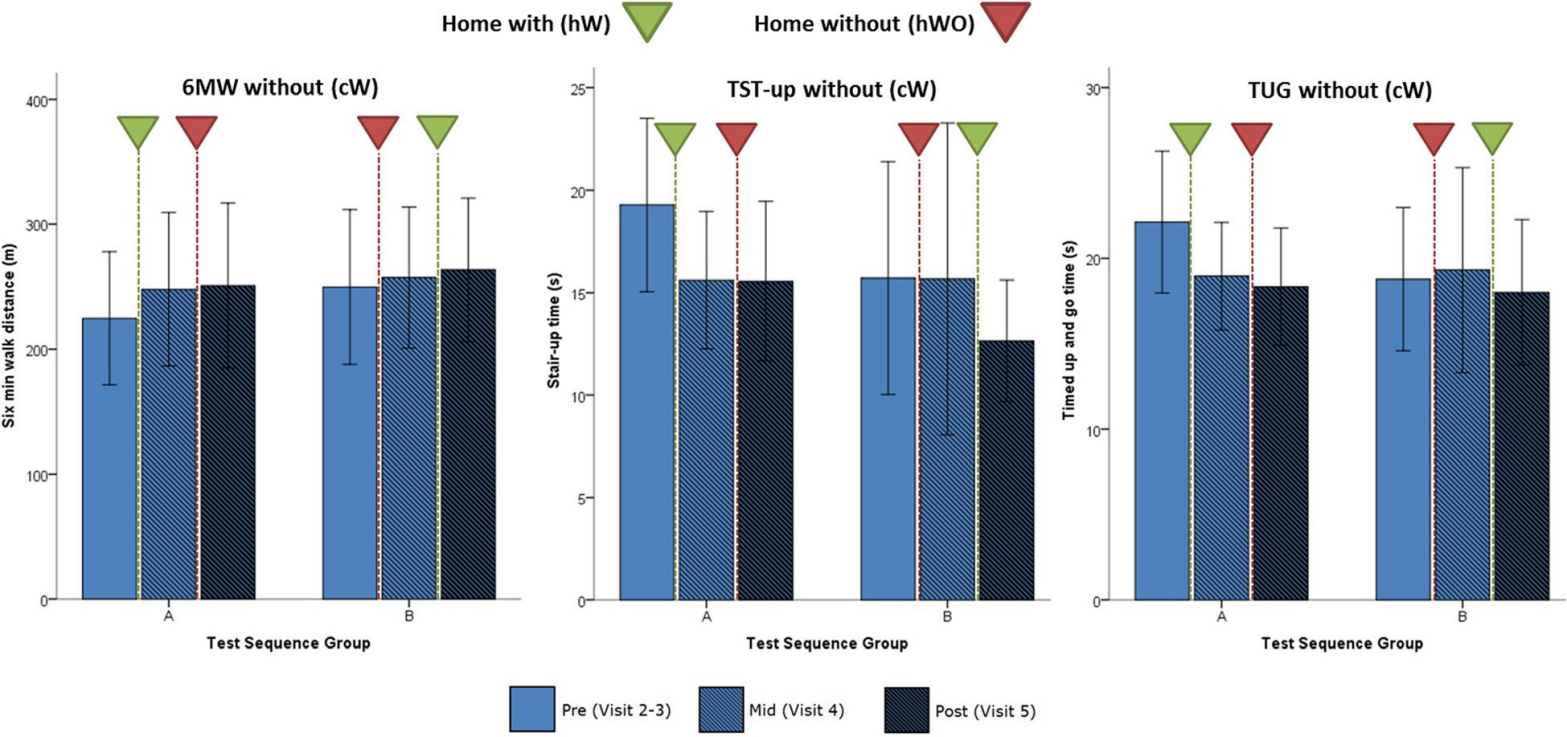
Training effect. TFT effects pre (light), mid-way (hatched), and post (dark) home trial, for 6 min walk (6 MWT) test, timed stair test (TST-up) and timed up and go test (TUG). Test results are with using Keeogo™ (cW). Home trial interventions are shown by the inverted triangle, where the green triangle represents 2-weeks home with Keeogo (hW), and the red triangle represents 2-week home without Keeogo (hWO). Error bars represent 95% confidence intervals. Results are shown for test sequence groups A and B (note the sequence of hW and hWO differed for A and B). In most cases the largest improvements were seen after 2-weeks hW

Using Keeogo at home might also impart improved skill and/or ability to use the technology. These “plastic” effects are akin to a plastic training effect. TFT data with Keeogo (cW) from participants’ visits before, mid-way, and following the two 2-week monitoring periods (hW,hWO) were used to quantify the training effect. These results are shown in Fig. 5. TFT scores for pre, mid and post-trial are shown for the cW testing condition, with inverted triangles showing the hW (green) and hWO (red) period between each measurement session.

##### 6 minute walk test

A mixed ANOVA was conducted for 6MWT scores between visits (visit: pre, mid and post) and cross-over group (group: A and B). The test yielded a significant visit effect (*p* < .001), non-significant group effects (*p* = .684) and a non-significant visit * group interaction (*p* = .237), indicating that test scores improved with each visit regardless of cross-over group. One-way ANOVA was then conducted within sequence groups. For group A, 6MWT scores increased significantly (*p* < .001) between pre and post measures. For group B, however, the increase was not significant (*p* = .274).

These results show that training with Keeogo in the clinic and home resulted in a small but measurable improvement in assisted 6MWT distance over the trial period of + 19.5 m. Although significantly different from zero (95% CI: 8.64,30.4) it was less than the measured rehab effect.

##### Timed stair test

A mixed ANOVA was conducted for TST up and down time between visits (visit: pre, mid and post) and cross-over group (group: A and B). For stair up time the test yielded a significant visit effect (*p* = .014), non-significant group effect (*p* = .448) and a non-significant visit * group interaction (*p* = .239), indicating that test scores improved with each visit regardless of cross-over group. One-way ANOVA was then conducted within sequence groups. For group A, TST-up score improved significantly (*p* = .017). For group B, however, the improvement was not significant (*p* = .349). Stair down time showed a similar result, with a significant visit effect (*p* = .007), non-significant group effect (*p* = .467) and a non-significant (but trending) visit * group interaction (*p* = .070). Follow-up ANOVA showed a non-significant pre-post change for group A (*p* = .033) and group B (*p* = .459).

The overall effect for stair performance was − 5.08 s improvement over the course of trial which was significantly different from zero (95% CI: -7.99,-2.17), and in this case, exceeded the MDC (− 3.47 s) for stair performance.

##### Timed up and go test

A mixed ANOVA was conducted for TUG test scores between visits (visit: pre, mid and post) and cross-over group (group: A and B). The test yielded a significant visit effect (*p* = .007), non-significant group effect (*p* = .650) and a non-significant visit * group interaction (*p* = .043), indicating that test scores improved with each visit. One-way ANOVA showed that for group A there was a significant decrease in TUG time between pre and post (*p* = .001), but for group B the improvement non-significant (*p* = .554).

Overall improvement in TUG performance was − 2.99 s which was significantly different from zero (95% CI: -4.69,-1.29), but did not exceed the MDC of − 3.5 s.

### Relationships between trial effects and usage

There were no significant correlations between performance effect, activity effect, rehab effect and training effect (*p* > .05) for any of the outcome measures.

The average daily use from the Daily Activity Logs was estimated to be approximately 2 h/day every other day (many patients tended to use it every second day, rarely did they use it every day). Usage scores on a 0–100 scale were 41.7+/− 20.13 for AMT, and 42.5+/− 20.5 for EXT, indicating usage was moderate. The Rehab effect correlated with the EXT (*r* = .452, *p* = .023) and the Training effect correlated with both AMT (*r* = .417, *p* = .043) and EXT (*r* = .438, *p* = .032). AMT and EXT were not significantly correlated (*p* > .05) with the immediate Performance effect or the home Activity effect.

### Adverse events

There were no serious adverse events related to Keeogo during the trial. The most common adverse events reported were skin irritation from cuffs, and muscle fatigue and soreness after exercise bouts. Although there was no planned analysis of falls, it is worth noting that of 6 falls reported by participants during the trial period, none occurred while wearing Keeogo. Although not reported as a fall, there was one curious circumstance where a participant became fatigued during a test session and after leaning and crouching against a wall, Keeogo slowly and deliberately lowered them to the ground and extended limbs leaving them prone on the ground. No injury was reported and the subject completed the home trial without further incident.

## Discussion

The purpose of this study was to evaluate the effects of the Keeogo exoskeleton on the physical performance of individuals with MS in a clinical setting, their physical activity levels in a home setting, and the exposure-dependent “rehab” and “training” effects.

Although a natural expectation of a robotically controlled powered exoskeleton would be to improve physical performance *while* the device is being worn, our data did not show this. Rather, participants with MS walked and negotiated stairs slower when wearing Keeogo, most notably when still naïve to the technology. Although participants demonstrated a significant improvement in their ability to use the technology over the course of the trial, and “closing the gap” between their performance measures with and without the device, on average they consistently performed slower on TFT testing at all visits when wearing the device.

This is not surprising, considering the device has mass and inertia to overcome, and has some inherent stiffness, which has been documented in a biomechanical study of healthy adults using the Keeogo [34]. Even though it was found in healthy subjects that gait speed did not differ significantly between trials when wearing and not wearing the Keeogo, the small difference that did exist (.06 m/s) when extrapolated to a 6MWTwould result in a distance deficit of − 21.6 m. This is approximately the deficit we saw for the performance effect (− 22 m) for patients with MS 6MWT when initially using Keeogo.

It may therefore be important that participants performed slightly “worse” while wearing Keeogo, given that the Rehab and Training effects were found to be significant and related to at-home use of Keeogo. This would suggest there is a positive physiological response in terms of muscle strength and/or endurance, possibly due to the low-level resistance and inertia of the Keeogo. Indeed, there is evidence that people with MS respond positively to resistance training [35]. The fact that at the same time Keeogo is ensuring movement assistance and stability indicates the device may have potential for delivering home and community-based exercise interventions.

This conclusion is bolstered by the finding that the Rehab and Training effects were correlated positively and significantly with exposure dose of Keeogo; the more that participants used Keeogo at home (which ranged from 0 h to > 30 h) the more their unassisted physical function improved, and the better they got at using Keeogo to perform physical activities.

Passive assist devices for the hip and ankle have been evaluated for patients with MS [36, 37]. Stationary robotic systems have been used with patients with MS ( [38], see review by [39]), and several studies have published findings from the ReWalk [40–42], Ekso [43, 44], Indego [45] and other over-ground exoskeletons for the stroke and spinal cord injury population. A review of these and other devices can be found in He et al. [11] and Lajeunesse et al. [12]. Synthesizing our findings with the literature is made difficult by the fact that there are presently limited studies that have examined a powered exoskeleton for people with MS [46].

A significant distinction that separates Keeogo from walking machines such as ReWalk, Ekso and Indego is that the latter devices are heavier powered hip and knee devices meant to be used in rehabilitation facilities for supervised gait training, and support the entire lower extremity and torso. Keeogo can be used in a clinical or home setting, only assists movements when required, and only powers the knee, thus weighs a fraction of the Ekso. As such it would be difficult to compare our findings to published findings on these other exoskeletons. Other exoskeleton suits more similar to Keeogo are on the horizon [47], but because their focus is on energy cost savings it is unclear whether the two technologies will be comparable.

In order to evaluate the rehab effects measured in the present study, we can look to a variety of randomized controlled trials (RCT) of physical activity/exercise training interventions on the MS population to compare our data. It is fortunate that the 6MWT is a very common outcomes measure for study of MS intervention effects. Using 6MWT change scores from published studies [5–9] the average change observed in these controlled intervention programs (+ 37 m) is not much different than the change that we observed (+ 28 m) for using Keeogo at home for 2 h/day every other day. Of note is that both values are well-below the computed MDC of + 65 m. This latter fact is troubling from a research design perspective and deserves some attention by the research community in this field.

Finally, there was little apparent impact on the amount of physical activity, as measured by step/day counts, when participants used Keeogo at home. This suggests participants benefited more from the exercise of wearing Keeogo rather than doing more physical activity with Keeogo. Also of note was that the average daily step count for the sample was 4425 steps/day, well under normative values (> 10,000 steps/day) for adults in their 40’s and 50’s, and more similar to healthy adults older than 65 [48]. Nevertheless, our finding was similar to values found by Dlugonski et al. [31], though less than values published by Sandroff et al. [32] and Motl et al. [33].

### Limitations

There are some important limitations of the trial. The trial was also relatively short. This was appropriate, however, given that the technology was new and unproven, and participant burden was relatively high, especially for the MS population.

We did not recruit participants according to MS phenotype (Relapse-remitting, Primary progressive, etc.) and therefore we are not able to analyze the findings according to these phenotypes. Future studies might consider this, however, we found that the participant’s physical condition was less a factor than their desire to use the technology. On the other hand, this could suggest that selection bias was a factor, whereby participants more interested in technology may be more likely to want to participate. The data showed a wide variety of usage (0–30 h) so we feel confident that selection bias was minimal, however future studies could inquire about technology attitudes among study participants.

The trial was designed to test the primary outcomes of an immediate benefit in performance (6MWT in the clinic and steps/day activity at home) while using Keeogo. Secondary outcomes were design to test for any rehabilitation and training effects that result from use of Keeogo over the trial period. Significant effects were only found for the secondary effects, but without a formal intervention during the at-home period, we cannot directly attribute the positive improvements in unassisted walking and stair climbing performance to Keeogo alone. A future clinical trial will be required to test for these effects.

## Conclusions

Although it was anticipated that Keeogo would benefit patients with MS by helping them walk faster and further, we discovered that its interaction with users was more complex. We conclude that Keeogo delivers benefits by enabling mobility assist and stability, while making patients with MS work a little harder when performing locomotor activities, thus having a net benefit on physical conditioning and capacity. More research is required to quantify these effects.

Also of interest was how well participants with MS tolerated the device. Despite their susceptibility to fatigue [13], MS participants were still able to benefit from Keeogo but they had to use the technology on a relatively consistent basis to see these benefits. Whether Keeogo is a suitable technology for every-day use is still to be determined, but as a tool for physical therapists it might offer possibilities for delivering exercise-based intervention to highly disabled populations that prior to now have not been feasible.
